# Assembling cheap, high-performance microphones for recording terrestrial wildlife: the Sonitor system

**DOI:** 10.12688/f1000research.17511.3

**Published:** 2021-02-19

**Authors:** Kevin Darras, Bjørn Kolbrek, Andreas Knorr, Volker Meyer, Mike Zippert, Arne Wenzel

**Affiliations:** 1 Department of Agroecology, University of Göttingen, Göttingen, Niedersachsen, 37077, Germany; 2 Celestion, Ipswich, Suffolk, IP6 0NL, UK; 3 Mess-, Steuerungs-, und Regeltechnik, University of Göttingen, Göttingen, Niedersachsen, 37077, Germany; 4 Konstruktion, Geräte- Neuentwicklung, Schreinerei, Schlosserei, University of Göttingen, Göttingen, Niedersachsen, 37077, Germany; 5 Functional Agrobiodiversity, University of Göttingen, Göttingen, Niedersachsen, 37077, Germany

**Keywords:** autonomous sound recorders, passive acoustic monitoring, signal-to-noise ratio, self-noise, acoustic horn, Song Meter, Swift recorder, Bioacoustic recorder, Audiomoth

## Abstract

Passive acoustic monitoring of wildlife requires sound recording systems. Several cheap, high-performance, or open-source solutions currently exist for recording soundscapes, but all rely on commercial microphones. Commercial microphones are relatively expensive, specialized for particular taxa, and often have incomplete technical specifications. We designed Sonitor, an open-source microphone system to address all needs of ecologists that sample terrestrial wildlife acoustically. We evaluated the cost and durability of our system and measured trade-offs that are seldom acknowledged but which universally limit microphones' functions: weatherproofing versus sound attenuation, windproofing versus transmission loss after rain, signal loss in long cables, and analog sound amplification versus directivity with acoustic horns. We propose five microphone configurations suiting different budgets (from 8 to 33 EUR per unit), and fulfilling different sound quality and flexibility requirements. The Sonitor system consists of sturdy acoustic sensors that cover the entire sound frequency spectrum of sonant terrestrial wildlife at a fraction of the cost of commercial microphones.

## Introduction

Passive acoustic monitoring of terrestrial wildlife is a firmly established field of study. It has many advantages over classical human observation methods ^1^ and bears considerable potential for further development ^2^. Birds, bats, amphibians, insects, and primates are often surveyed using autonomous sound recorders. A wide range of open-source devices and commercial products exists for recording sound in terrestrial habitats ^3^: Established manufacturers offer products to cover all needs, and non-profit organisations also build and sell autonomous sound recorders. Raspberry-Pi based solutions, as well as dedicated, open-source automated sound recorders offer cheap “do-it-yourself” alternatives to commercial products.

Microphones, as transducers of mechanical energy into electrical signals, are the most important component of a sound recorder. They are the first step in the sound recording process, and through their frequency response, they determine which animals can be recorded. Using a literature meta-analysis and an experimental approach, we recently demonstrated the crucial importance of microphone specifications and underlined how microphone signal-to-noise ratio, a measure of its inherent noise level, affected the sound detection space (i.e., its detection range) ^4, 5^, which is also determined by external factors ^6^.

Despite the many different sound recorders that are available, their owners are usually restricted to the microphones of the respective manufacturers or the recommendations of recorder builders due to compatibility or warranty issues. However, outdoor microphones rapidly degrade as they are exposed to rain ingress, animal damage, ultraviolet radiation, and wide temperature ranges ^7^. Thus, they need to be replaced often, but end-users can usually only buy expensive replacements from the original manufacturer as repair instructions are not available, components are unknown, and the design is not disclosed or even protected against inspection. Microphone specifications are rarely complete, usually only stating sensitivity instead of the more informative signal-to-noise ratio. In many cases, the microphone element that is used is unknown. In some cases audible sound frequencies (i.e., frequencies below 20 kHz) are filteredinside the microphone to enable only bat recordings although the underlying microphone elements are capable to record the entire acoustic spectrum from amphibians to bats (Wildlife Acoustics, e.g. SMX-U1, SMM-U2 microphones). Currently, no external microphone is available to record both bats and birds, although the recorders that can record ultrasound theoretically could sample both audible sound and ultrasound. Note however, that the Audiomoth recorder has an integrated microphone element that samples sound up to 192 kHz ^8^.

To provide alternatives to the sound-recording community of ecologists, we designed a cheap, open source, high-performance, and modular microphone system called Sonitor. We first present the basics of microphone components. Then, we show general constraints of microphone design: We measure trade-offs between weatherproofing and transmission loss, between wind-proofing and drying time, between cable length and signal loss, and between directivity and analog amplification. We test different acoustic vents and put different microphone protection strategies to the test in a long-term, outdoor durability test. Based on our results, we discuss aspects of microphone protection and sound quality, and we present the general design of Sonitor microphones, along with 5 concrete microphone types that can be used for different use cases to record all terrestrial wildlife. We evaluate the temporal and financial cost of the assembly and detail their compatibility with recorders.

## Methods

### Microphone design basics

Sound consists of pressure waves travelling through a medium, in our case air. Human-audible sound makes the air vibrate at frequencies between 20 Hz and 20 kHz. Ultrasound, which is not audible for us, extends beyond 20 kHz. Insects and bats can emit and perceive ultrasound up to 200 kHz ^9^. Microphones are transducers of mechanical energy (pressure waves) into electrical energy (a voltage). A variable voltage is created as sound waves move mechanical parts of microphones, which can be a polarized membrane (electret condenser), or a piezoelectric element. There are mainly two types of microphones used in autonomous sound recorders: electric condenser microphones (ECM capsules) and microelectro-mechanical systems (MEMS) microphones. The older ECMs use only two terminals and require a voltage bias to operate ^10^; they have relatively large diaphragms with more inertia and thus inherently respond weakly to high sound frequencies such as ultrasound, MEMS microphones use three terminals and do not require bias voltage; they are usually sensitive to ultrasound. The role of the recorder is mainly to increase the minimal voltage differences with amplifiers, digitize them with analog-to-digital converters, and record them to a digital storage medium (mostly solid-state memory, secure digital cards).

Outdoor microphones are electrical devices which need to be protected against water ingress, and climatic and mechanical shocks. Protection comes from solid housings, often metal tubes in which the microphone element is inserted. The microphone element (often ambiguously called simply "microphone") is the centerpiece of the microphone and consists only of the acoustic sensor which transduces sound to a variable voltage. However, microphone housings need to be open to allow sound to reach the microphone element through their acoustic port. Since an opening would allow water to penetrate the microphone, corrode its components, and block the sound path, protection is needed. Acoustic vents are used for microphones that are not explicitly protected against ingress: they are transmissive for sound while being impermeable to water or hydrophobic, and thus fulfil a crucial function for outdoor microphones. Further, microphones need to transmit their output voltage to a recorder via electrical wires. When microphones are interchangeable, they use an audio connector as interface, which needs to be weather proof too.

Basic microphone properties can be augmented with attachments. Windscreens, usually made of synthetic foam or fur, reduce unwanted wind noise which comes from friction of air against the microphone. They also reduce potentially damaging water pressure from rain drops. Furthermore, parabolic reflectors or horns can be used to gather sound over a larger area before concentrating it to the microphone element, but the gained amplification is traded off against higher directivity: the sound pickup pattern becomes narrower.

### Microphone components

***Microphone element***. We chose to use mostly MEMS microphones due to their high performance at small sizes, the potential of that newer technology to offer higher performance gains than conventional ECM capsules, and finally their lower part-to-part variation and sensitivity to temperature variations ^11^. Different elements exist that can fulfil different requirements by prioritizing low-noise recording, a wide frequency response, or weatherproofing. We are using microphone elements from different manufacturers. We used a tried-and-tested element from Knowles (SPU0410LR5H-QB), which was used by the company Biotope.fr inside the now discontinued BIO-SMX-US microphone as a substitute for SMX-US microphones by Wildlife acoustics. We also used it inside our own housings since 2017 for recording birds and bats with SM2Bat+ recorders. We tested Invensense's ICS-40720 element, which features low-noise recording (specified signal-to-noise ratio of 70 dB) and also Vesper's VM1000, which is a piezo-electric element that is waterproof and resistant to various environmental stresses. All three MEMS elements have a typical sensitivity of -38 dB and thus require relatively strong amplification from the recorder for soundscape recordings. Note that the Audiomoth recorder features microphone elements with built-in amplification (Knowles SPM0408LE5H-TB). Large ECMs usually are more sensitive due to their bigger diaphragm (Primo EM172: -28 dB, used in Solo recorder, BAR and presumably in SMM-A2 microphone by Wildlife Acoustics).

***Printed circuit board (PCB)***. Microphone elements can be directly soldered to cables, but this requires great care and dexterity for a precise and rapid soldering that does not exceed the temperature tolerance of the element (this was done for SMX-US microphones, Wildlife Acoustics). Moreover, a precise alignment of the microphone within the housing and with the acoustic vent is needed for compatibility with external attachments and for enabling consistent part-to-part quality. It is thus preferable to reflow-solder MEMS elements to printed circuit boards. This can be performed in reflow oven equipped electronic laboratories or workshops. This is readily available as a paid service and is a burgeoning business satisfying the needs of electronic equipment manufacturers and electronics hobbyists in need of prototypes. Cables can then be more easily soldered to PCBs without damaging the microphone element. The microphone and conductive tracks can be attached on the bottom side of the PCB, which guarantees a result that is flush with the housing. PCBs can be ordered in any size and shape with a variety of support materials. For the larger ECMs, manual soldering is less challenging, so that they do not have to be combined with PCBs.

***Housing***. The microphone elements are preferably held by simple metal tubes, and the wiring is inside. The housing can be made out of stainless steel or lighter aluminium, these metals offer high resistance to weather and mechanical shocks, are cheap and readily available, and easy to glue. They can also be painted to reduce their visibility in natural environments. Due to their hardness, metals can also be machined with high precision to ensure stable results within tight tolerances so that any attachment can easily fit the housing.

Alternatively, the audio connector itself can be used to house the wiring, and ECM elements can be glued directly on top of it, which leaves them exposed to environmental stressors.

***Wires and connector***. We chose standard 30 AWG stranded wires for more flexibility compared to solid wires. At one end, the cables are connected to the PCB, which is connected to the microphone element. At the other end, the wires are connected to Mini-Con-X series waterproof connectors. The connector’s backshell and grommet, which is needed to release the tension when the connector is attached to flexible cables, can be omitted when using metal housings. Mini-Con-X connectors are commonly used in most autonomous sound recorders (Wildlife Acoustics and Frontier Labs recorders, Swift, Arbimon). They can withstand some abuse and are ingress-protection rated at IP67 (dust tight and protected against water up to 1 m deep).

***Acoustic vent***. We use acoustic vents to protect the non-waterproof microphone elements against solid and liquid ingress. Currently, we use different products in varying sizes and protection levels against water that are available from Gore, but we tested vents from other manufacturers as well. The Gore GAW112 vents can be used; they appear identical to the ones used in SMX-US, SMX-U1, and SMX-II microphones from Wildlife acoustics. They need to be coupled with windscreens, as GAW112 vents let water pass after immersion or drop projection. We also tested GAW325 vents, which are IP67 rated. Freshwater ingress *per se* only temporarily blocks microphone elements that are not waterproof from vibrating, but will not short-circuit the microphones due to the low conductivity of freshwater. However, water leads to corrosion, which will destroy microphones and conductive tracks, given enough time. The GAW3XX series also have a support material, which can be made of woven or non-woven PET material. The PET (woven) support elements are better suited as they absorb water less.

### Acoustic assessment

All assessments of the microphones’ technical qualities were performed with SM2Bat+ recorders (Wildlife acoustics), which allow to record two channels simultaneously up to a maximum sampling frequency of 192 kHz. We measured sound across three frequencies that can roughly be assigned to different taxa: 1 kHz (birds and amphibians), 10 kHz (insects), and 40 kHz (bats). We used a battery-powered one-driver Anker SoundCore loudspeaker for emitting audible pure test tones at 1 and 10 kHz (generated using Audacity 2.2.2) and an ultrasonic calibrator (Wildlife Acoustics) that emits chirps at 40 kHz. Since we did not have access to dedicated anechoic rooms, all tests were conducted outdoors, and we also refrained from using test tone frequencies below 1 kHz, as lower-frequency anthropogenic noise was constantly present. Test sounds were emitted to the front of the microphones and when needed also to the side at a 45° or 90° angle. We generally measured the amplitude of ten ultrasound chirps (0.7 s total duration) and three 0.8 s test tones (2.4 s total duration) for the 1 and 10 kHz frequencies in recordings with a sampling frequency of 96 kHz in Audacity, by exporting the frequency spectra with a Hanning window size of 1024 and choosing the frequency window that included our tone's base frequency.

***Weatherproofing vs. sound attenuation***. The only point that is permeable to sound is the acoustic vent, and its permeability to water ingress is given by its IP (Ingress Protection) rating. The sound attenuation at 1 kHz is usually also indicated in the product specifications given by the manufacturer in decibels (dB), as this is the frequency most relevant for recording human speech. However, terrestrial wildlife sounds span frequencies from 20 Hz to 200 kHz.

**Figure 1.**
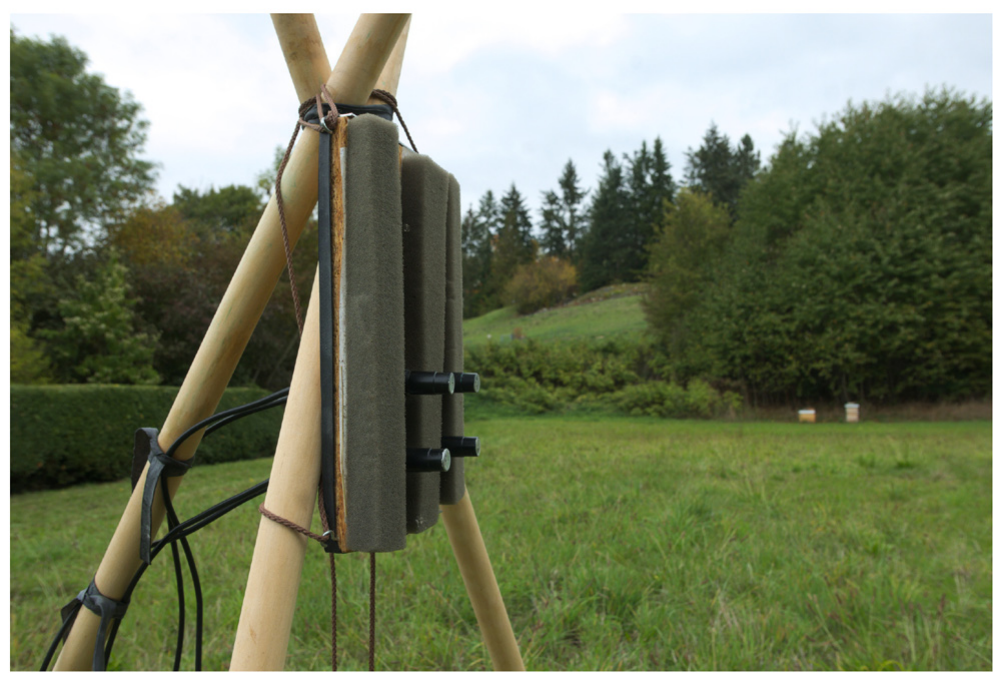
Setup used for testing microphone attachments outdoors. The microphones were approximately 1 m above the ground and parallel to each other.

We compared sound attenuation or potential amplification of 2 GAW112 (inner diameter of 3 mm) and 2 GAW325 (inner diameter of 2.4 mm) vents (for article version 2; Gore, U.S.A.), as well as three vent models from different manufacturers (for present article version; 1: SV-021 ePTFE vent, Sinri, China; 2: LY-WBAMG-085 PU vent, LiYue, China; 3: ”TK” ePTFE vent, Thomas Kore, China) with an open setting without vent, outdoors ( Figure 1). We recorded the US calibrator and loudspeaker tones at 3 m from the microphones, to the front and at a 90° angle to the side. For article version 2, four Knowles SPU0410LR5H-QB microphone elements, and for the current version, four Vesper VM1000 microphone elements were used, and we re-tested the GAW325 vent. Microphone elements were reflow-soldered behind a 1 mm hole in a 1 mm thick PCB. We used the microphones open, and pasted the vents onto them for testing the change in sound transmission. We calculated the relative attenuation of the vents for each microphone unit by subtracting the absolute sound level of the open microphone from its sound level with the vent attached, and we compared the mean attenuation to zero by calculating 95% confidence intervals.

**Figure 2.**
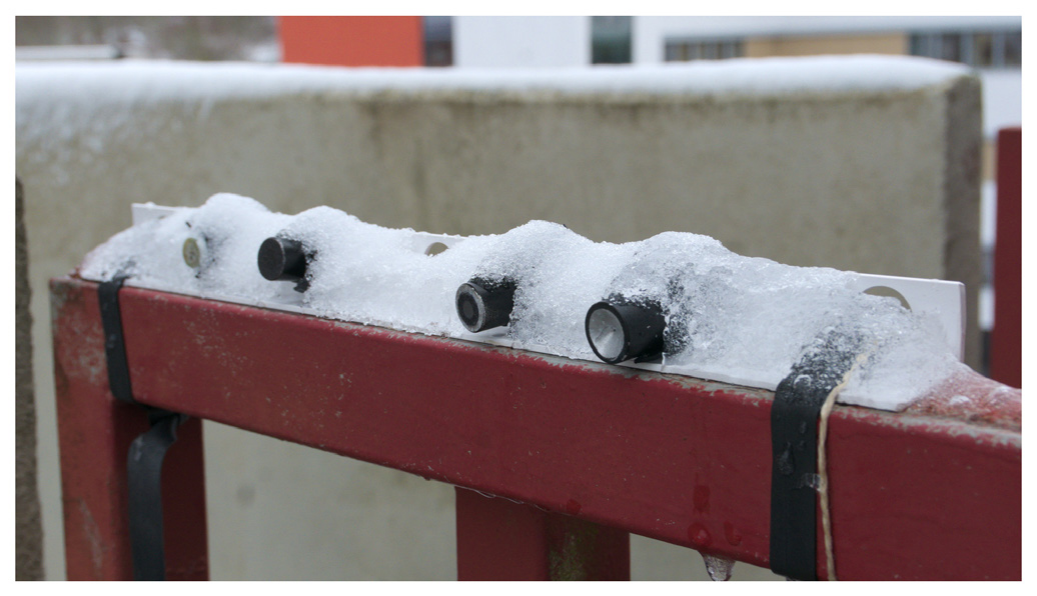
Different microphone prototypes exposed to outdoor conditions during winter 2018 in Germany.

**Figure 3.**
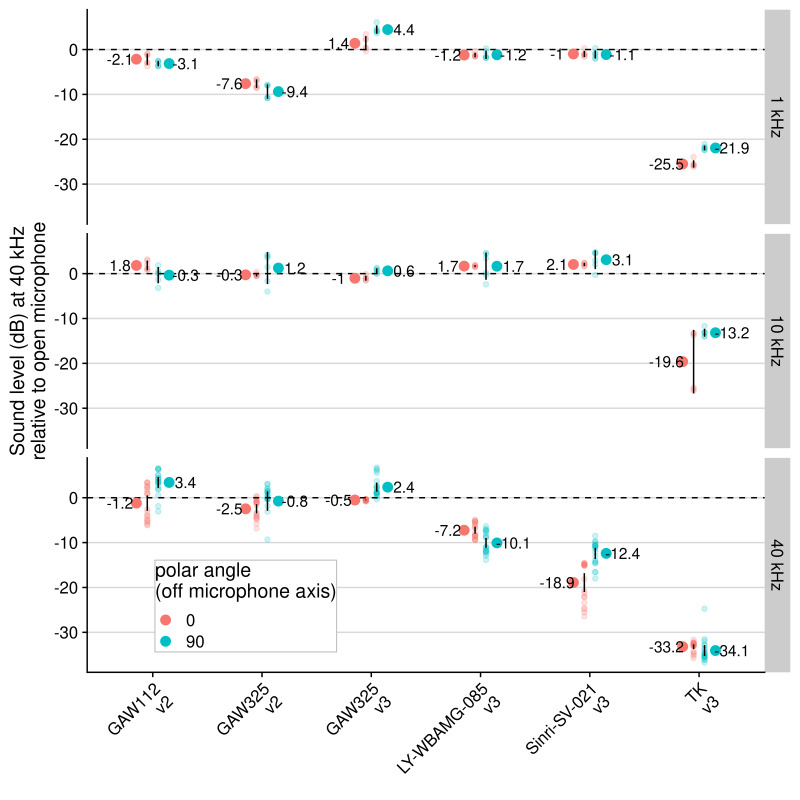
Sound transmission loss caused by five different acoustic vents, in front and to the side of the microphone axis, shown with 95% confidence intervals. The GAW112 offers IP4X to IP6X protection, the LY-WBAMG-085 and SV-021 offer IP67 protection, te “TK” vent has a non-rated protection level, and the GAW325 vent offers IP67 to IP68 protection (ratings depend on the housing).

Additionally, to check whether microphones still work after exposure to water, we immersed one Knowles microphone, one Vesper microphone and one Invensense microphone into a plastic recipient filled with distilled water. We recorded music and ultrasound chirps before, during, and after immersion. We checked whether the immersion destroys the microphone elements or affects the sound recording qualitatively.

***Windproofing vs. drying after rain***. We used Knowles SPU0410LR5H-QB elements outdoors; one was protected by a GAW112 vent and a windscreen (Wildlife Acoustics), one had a 6 mm long horn attached (see article version 1), and one had a GAW325 vent. All three configurations represented similar levels of water ingress protection, but we used the Knowles SPU0410LR5H-QB with the 6 mm horn instead of the Vesper VM1000 (for which it was designed) to equalize the microphone model. We recorded test sounds from the loudspeaker and the calibrator at a distance of approximately 4 m. We placed a 62 W fan at approximately 30 cm from the microphones, to the front and to the side to simulate wind. We recorded the test sounds to check how prone to noise the vent-only and horn-only microphones are in comparison to the microphone with the windscreen. Then, we drenched all microphones in distilled water to simulate heavy rain. We continued recording test sounds immediately after, as well as 1, 3, 18, and 66 hours after the simulated rain to check how long sound transmission was attenuated by the different wet attachments. We measured the sound level of the 1, 10, and 40 kHz tones recorded by each microphone relative to the sound level recorded after 66 hours of drying.

***Cable length vs. signal loss***. Microphones usually advertise built-in amplifiers to strengthen the relatively low voltage signals of the microphones so that they do not degrade over long cable distances. High frequencies are more prone to signal degradation because the capacitance of the cable causes more attenuation at high frequencies. We tested whether the output signals of the Knowles SPU0410LR5H-QB microphones were affected by long cables, which are sometimes needed for installing microphones far apart or in different locations than the recorders themselves. We attached two Knowles SPU0410LR5H-QB microphones to the recorder, one via a 5 m cable and the other one via a 52.5 m long cable. They were close to each other and pointing in the same direction. We recorded test sounds emitted with the loudspeaker and the ultrasound calibrator at 6 m from the recorder. We recorded the same test sounds after switching the cables to check whether the results were driven by the microphone itself. We measured 20 ultrasound chirps for each microphone with each configuration.

***Directivity vs. amplification***. We built horns for amplifying the acoustic input signal before it is transduced by the microphone. Horns increase signal-to-noise ratio and ultimately lead to greater detection ranges. However, acoustic horns are generally directive: At high frequencies, horns will mainly respond to sounds within their opening angle, where direct sound can reach the throat of the horn. Outside the opening angle, low-frequency sounds reach the throat of the horn by diffraction.

The reasoning behind using horns is that in stereo deployments, there is a redundancy of recorded data: omnidirectional microphones pointing in opposite directions are recording much of the same data twice. To make better use of them, one can use acoustic horns that amplify the sound from the front and decrease sound from the back or the sides. Ultrasound, which propagates less far, benefits especially from horns, because even very small horns can achieve considerable amplification. For ultrasound, horn dimensions can also be held almost as small as the existing microphone housings. Also, microphones usually suffer from a drop in the frequency response and/or signal-to-noise ratio in the ultrasound range, thus horns help to attain a desirable, more linear frequency response.

 We chose a horn design that has steadily increasing amplification with frequency starting approximately from 10 kHz and minimal directivity. Conical horns are generally more suitable than exponential horns, which do not amplify sound much above a certain threshold. Horn dimensions were chosen by calculating and simulating the theoretical analogue amplification in-axis and off-axis using numerical methods to choose the most favourable designs. The gain of the horns was calculated using one-dimensional equations for conical horns ^12^. Since the one-dimensional calculations could not predict directivity, Boundary Element Method models ^13^ were set up to model the directivity of the horns. The ultimate gain depended mainly on the ratio of the areas between the mouth and throat of the horn, while the frequency range depended on the length of the horn. A long and narrow horn will also be resonant, which will increase the gain but reduce the fidelity of the recorded sounds.

Previously (article version 1), we investigated whether different ultrasonic horns could amplify the signal enough to compensate for the transmission loss due to the acoustic vents. The Knowles SPU0410LR5H-QB and Invensense ICS-40720 microphones require the use of the GAW112 or GAW325 vents for ingress protection. When pasted onto the horns, the diameter of the vents’ active surface (through which sound travels) dictates the maximum mouth diameter and theoretical amplification of the horn. Even though we chose the largest vents available, the resulting horns were too small to offset the ultrasound transmission loss incurred by the use of the vents (Figure S1, article version 1). In the previous article version, we also tested how much amplification could be gained with different horns placed in front of the Vesper VM1000 elements, which do not require vents and allow for a larger horn mouth diameter. Still, we decided to constrain the horn dimensions to limit the resulting diameter of the microphone. We found that the longer the horns, the higher the achieved transmission, but the losses for sounds coming from the side also increased, as the horns were more directional (Figure 5, article version 1).

**Figure 4.**
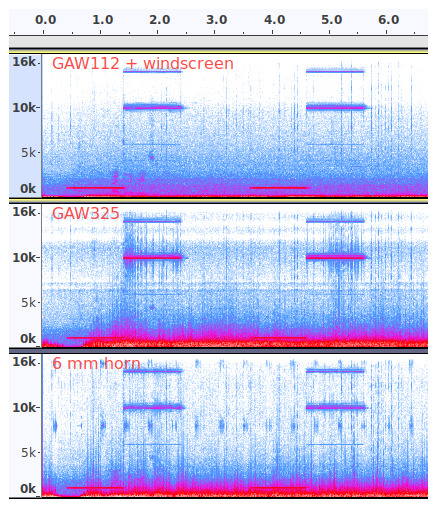
Spectrograms of different microphone designs (GAW112 and GAW325 are acoustic vents) showing wind noise. Without windscreen, 1 kHz test sounds are masked by wind noise.

 These experiments prompted us to increase the overall dimensions of the ultrasonic horn to increase the amplification and to make it less directive. Our calculations resulted in a horn with a mouth of 20 mm, a throat of 1 mm, and a length of 5 mm, resulting in a half angle of approximately 62°. We chose to place the acoustic vents at the horn throat to be free to design horns with large mouth diameters, but we kept the overall horn diameter at a maximum of 2.1 cm for practicality purposes. We measured the ultrasound amplification of the horn along three axes (0°, 45°, and 90° angle off the microphone axis) at 6 m (due to the higher amplification). We could not calculate/simulate the effect of the acoustic vent on the amplification of the horns so we decided to measure the horn amplification when used with and without a GAW112 vent at its throat.

We also tested horns for audible sound. As the lower limit of the amplification of a horn depends on its size compared to the wavelength, they had to be much bigger than the ultrasonic horns. They were therefore made out of PVC to keep the weight low, with a mouth of approximately 15 cm, a throat of 5 mm, and a length of 10 cm (resulting in an angle of 90°).

***Durability test***. We exposed four different microphone prototypes, representative of our Sonitor designs presented below, to outdoor climatic conditions from central European winter until summer to test their durability ( Figure 2). We used three microphones with a VM1000 element, either bare, or protected with a GAW112 vent, or protected by a 3 mm long horn. We used one microphone with a ICS-40720 element, protected with a GAW325 vent. We used a microphone with a SPU0410LR5H-QB element without an acoustic vent as a reference microphone that was kept indoors at room temperature (ca. 20 °C) between recordings. We installed the prototypes at the north face of our research group’s building (WGS 84 geographic coordinates: 51.559006, 9.953170) from November 23, 2018 until August 16, 2019. We recorded test tones at 1, 10, and 40 kHz on six different occasions, with a recorder amplification of 36 dB. The position of the sound emitters and microphones did not change. Test tone sound levels were measured in Audacity and then standardised by subtracting the sound level of the first recording to visualise the relative sound level change. We also measured the ambient noise – which includes the microphone self-noise – between test sounds. We computed the signal-to-noise ratio of the different microphone designs by subtracting the ambient sound level from the signal sound level and plotted it against time to compare their protection levels. This allowed us to consider changes in microphone sensitivity relative to their self-noise.

## Results

### Weatherproofing vs. sound attenuation

Vent models had variable qualities, but ultrasound was attenuated more than audible frequencies, and the attenuation depended slightly on the sound direction ( Figure 3). The GAW112 vent attenuated 1 kHz sounds (front: -2.1 dB, side: -3.1 dB), amplified 10 kHz sounds from the front by 1.8 dB, and amplified 40 kHz sounds from the side by 3.4 dB. The GAW325 vent, when tested on the SPU0410LR5H-QB (article version 2), attenuated 1 kHz sounds (front: -7.6 dB, side: -9.4 dB) and attenuated 40 kHz sounds from the front by -2.5 dB. The GAW325 vent, when tested on the VM1000, amplified 1 kHz sounds from the side by 4.4 dB, attenuated 10 kHz sounds from the front by 1 dB, and amplified 40 kHz sounds from the side by 2.4 dB. For both GAW vents there was a tendency of amplified ultrasound from the side. The LY-WBAMG-085 vent attenuated 1 kHz sounds by 1.2 dB both from the front and side, amplified 10 kHz sounds from the front by 1.7 dB, and attenuated 40kHz sounds (front: -7.2 dB, side: -10.1). The Sinri-SV-021 vent attenuated 1 kHz sounds (front: -1 dB front, side: -1.1 dB), amplified 10 kHz sounds (front: 2.1 dB, side: 3.1 dB), and attenuated 40 kHz sounds (front: -18.9 dB, side: -12.4 dB). Lastly, the “TK” vent attenuated sounds at 1 kHz (front: -25.5 dB, side: 21.9 dB), 10 kHz (front: -19.6 dB, back: -13.2 dB) as well as at 40 kHz (front: -33.2 dB , side: -34.1 dB). Data for the vent transmission loss are available on OSF ^14^.

All three types of microphones were able to record sounds, albeit distorted, under water. Once the water droplets that accumulated in the acoustic ports of the microphones were shaken off, all microphones continued to record normally after immersion in distilled water ^14^.

### Windproofing vs. drying after rain

The windscreen significantly reduced wind friction noise ( Figure 4). The vent-only and 6mm horn configurations were affected by wind friction noise at up to 3 kHz, greatly masking the 1 kHz test tones, although they were still audible and visible in spectrograms. However, detectability of target sounds below 1 kHz should be even more negatively affected.

**Figure 5.**
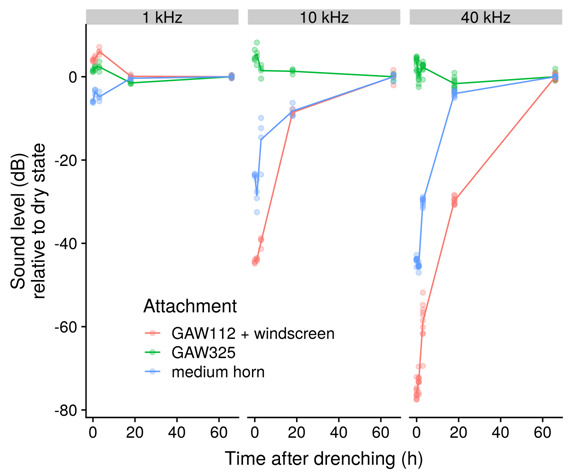
Recorded sound levels with drying time for different microphone configurations (GAW112 and GAW325 are acoustic vents) at different frequencies.

The GAW112 vent with windscreen combination needed much longer to dry than the 6 mm horn ( Figure 5). When still wet, one to three hours after drenching, high audible frequencies (10 kHz) were attenuated around 20 dB and ultrasound around 30 dB more than the 6 mm horn ( Figure 5). After at most 18 hours, the droplet that could have blocked sound from reaching the microphone acoustic port had evaporated and the microphone recorded sound levels as high as when entirely dry. Low audible frequencies (1 kHz) were not impeded even by water-logged windscreens. The waterproof, hydrophobic GAW325 vent ensured that no water blocked the sound path: sound of all frequencies were recorded at approximately the same level, irrespective of the time after drenching. Data for the drying experiment are available on OSF ^14^.

### Cable length vs. signal loss

We found that the 52.5 m cables decreased the sound level of our 40 kHz test chirps by 1.2 to 1.3 dB compared to 5 m cables. Data for signal loss with increasing cable length are available on OSF ^14^.

### Directivity vs. amplification

**Figure 6.**
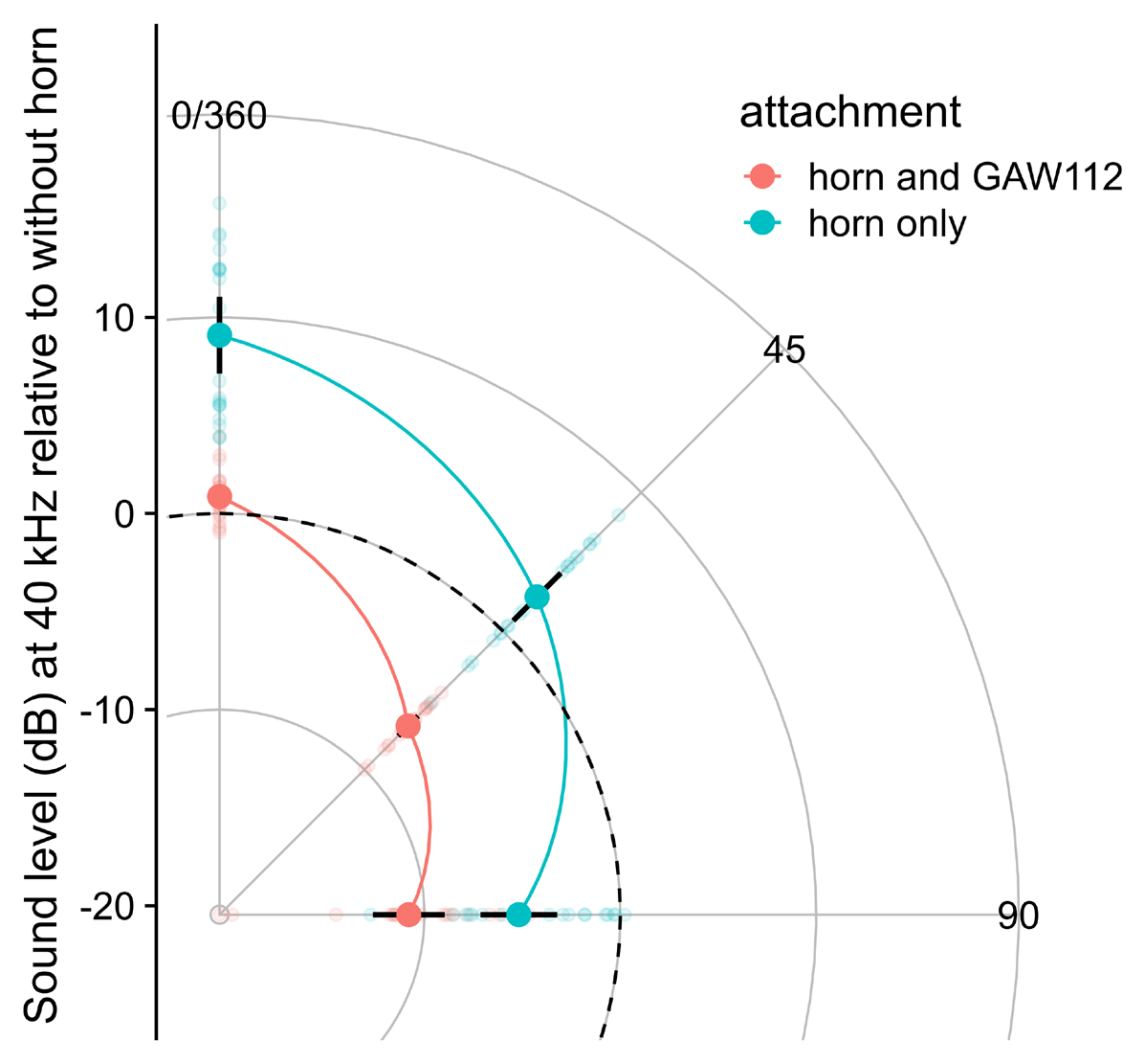
Partial polar diagram of the sound level amplification obtained with the ultrasonic horn for sounds coming from different angles (in degrees) to the microphone axis. The horn amplification was tested with and without a GAW112 acoustic vent placed at its throat.

The ultrasonic horns amplified sound from the front by 9.1 dB, amplified sound from 45° by 2.5 dB, and attenuated sound from 90° by 5.2 dB ( Figure 6). The gain was smaller and directivity higher than simulated, probably due to diffraction. When combined with the GAW112 vent at its throat, the horns amplified sound from the front by 0.9 dB, attenuated sound from 45 degrees by 6.8 dB, and attenuated sound from 90 degrees by 10.8 dB. The data are available on OSF ^14^.

 Our large acoustic horns amplified sounds from the front by 3.4 dB at 1 kHz and by 7.2 dB at 10 kHz. This was less than predicted by the numerical calculations, possibly because of interactions between the acoustical impedance of the horns and the mechanical vibrating system of the microphone. This interaction had not been taken into account in the calculations, as the mechanical constants of the microphone were unknown.

### Durability test

**Figure 7.**
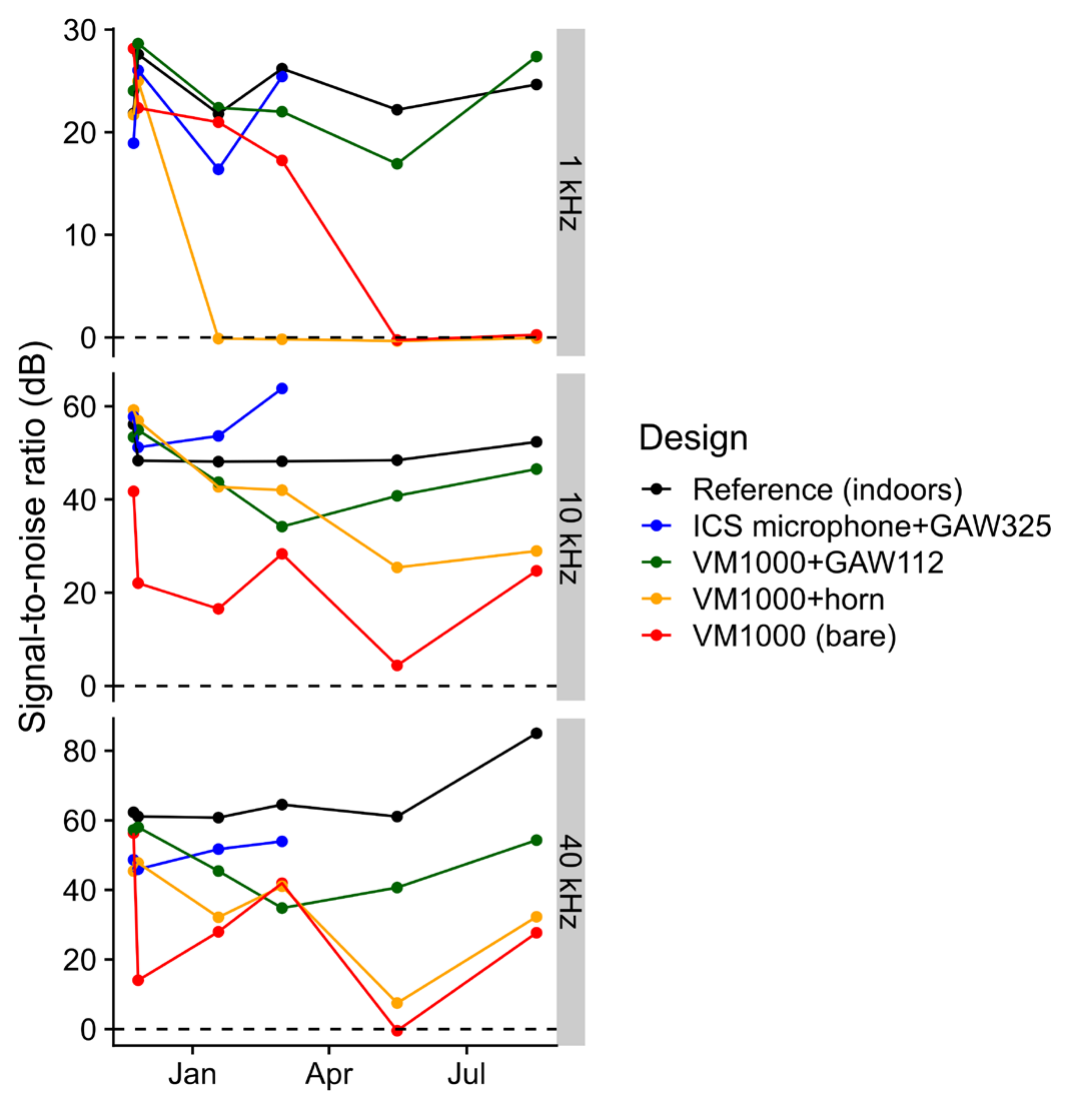
Change of signal-to-noise ratios of microphones protected with different attachments (GAW112 and GAW325 are acoustic vents) at different sound frequencies. Signal-to-noise ratio here is defined as the difference between the ambient sound level and the signal sound level. The acoustic GAW325 vent that protected the ICS-40720 microphone was pierced after the recording in March.

The signal-to-noise ratio of the reference microphone was the most stable ( Figure 7) with relatively constant ambient and signal sound levels. The microphone that was protected by the GAW325 also had a stable signal-to-noise ratio but it was damaged after the third point in time (the cause is unknown). In comparison, all other designs had more variable signal-to-noise trends in time. The VM1000 designs that were not protected by acoustic vents had even more variable signal-to-noise ratios that generally decreased with time, and they reached zero at 1 kHz.

## Discussion

**Figure 8.**
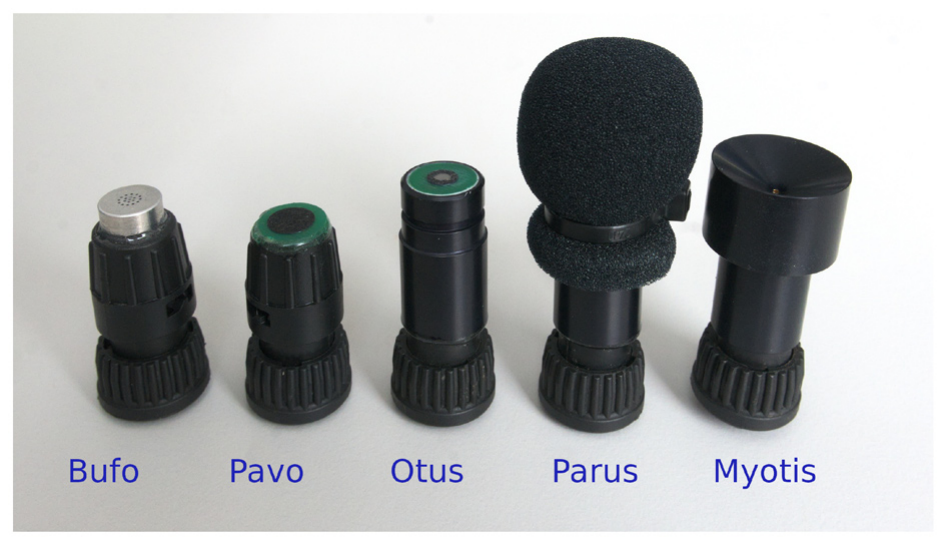
The current Sonitor microphone models. Note that the Bufo does not have a protective vent on this photograph. The Parus has a windscreen attached with a cable tie, and the Myotis is depicted with the ultrasonic horn.

The best microphone configuration will depend on the organisms of interest, the presence of wind and rain, and the need for directional sound recording. Many different designs are possible, all of which have not been tested or built here. Our results demonstrate that no design is perfect and shows how desirable properties trade off against disadvantages. Here, we discuss microphone protection and sound quality aspects. Then, we present five microphone designs offering different balances of positive and negative characteristics, optimised for specific use scenarios, and named after representative genera ( Figure 8).

### Protecting microphones against water and wind

Overall, protecting microphones against water ingress and mechanical damage while ensuring maximal sound transmission is a challenge. Different combinations of vents of different grades and windscreens help attain satisfying protection levels, however they all come with some drawbacks that are best analysed in the light of each study’s context. Most manufacturers couple acoustic vents like the GAW112 with windscreens to achieve high protection levels against water ingress and wind noise (e.g. Wildlife Acoustics microphones). In habitats or regions with little wind, especially for avian studies, it becomes worthwhile to use only high-performance vents like the GAW325, thus avoiding sound transmission losses when windscreens are drenched with water after rain. However, in some cases it can become necessary to protect these vents with a metal grid, as the acoustic vents can get pierced: We observed this in our own test and Wildlife Acoustics issued an advisory statement to say that birds can mistake the vents for a flower (presumably because of the two-color circular appearance) and pierce it so that all SMM-U2 microphones had to be upgraded with additional protection.

For recording bats, high degrees of protection with acoustic vents can come at the expense of ultrasound transmission. The attenuation depends strongly on the exact microphone design, the microphone element itself, and the vent used. The attenuation of the waterproof Gore vent (GAW325) when held by a vent holder or horn (article version 1) was much higher than when pasted directly onto the PCB, indicating that a large air gap behind that vent may be detrimental for sound transmission. It appears that the GAW325 also muffles ultrasound when used in combination with the Knowles element, but there was no detectable attenuation when combined with the piezo-electric Vesper element. Finally, three of our most recently-tested vent models had unacceptable levels of ultrasound attenuation, above 7 dB (which would entail a halving of the detection range). The usual approach with unprotected microphone elements would be to use GAW112 vents with windscreens: ultrasound is not detectably attenuated by the GAW112 vent. However, windscreens are not needed for bats because wind noise only reaches frequencies around 3 kHz: Wildlife Acoustics forewent the decision to include windscreens on their latest ultrasonic SMM-U2 microphone. Moreover, drenched wind screens block ultrasounds much more than audible frequencies. Thus, a sensible approach would be to use waterproof elements like the VM1000, coupled only with a GAW112 vent that prevents droplets to block the acoustic port, when ultrasonic horns are not needed.

It is also possible to reduce the rain ingress by blocking it with shelters or solar panels ^15^, although this could block sounds of interest coming from above the microphone to some extent. Interestingly, since all microphones are able to record sounds underwater and record normally thereafter, the Vesper VM1000 microphone seems to attain waterproofing only because of the tight solder pattern around the acoustic port, which prevents water to get inside the housing.

 According to our durability test, protecting microphones in the long term against outdoor elements is challenging. It was already shown that microphone sensitivity can degrade with time ^7^, but we found that exposure to climatic elements both increases the self-noise level and decreases the sensitivity ^14^, resulting in stronger decreases in signal-to-noise ratio ( Figure 7). Surprisingly, microphones such as Vesper’s VM1000, even though specified as waterproof, are not necessarily usable outdoors for extended periods of time without protection. Water drops can block the acoustic port (which may have caused the rapid signal-to-noise ratio drop at the beginning of the test), temperatures vary over large ranges, animals can enter the microphone, dust and pollen can fly in, solar radiation can rapidly heat the electronics, rain can ingress, and ice can form within the microphone, which would almost certainly destroy the microphone due to its expansion. However, the VM1000 uses a different (piezo-electric) design and it is conceivable that it should be more resistant than classical MEMS microphones using condenser membranes. Indeed, the normally unprotected VM1000, when additionally equipped with a simple GAW112 acoustic vent, could still record all the test frequencies after almost 6 months outdoors, which is an atypical and strenuous test with temperature spanning a range of 40°C. Therefore, we recommend to always use acoustic vents to protect microphones, except when using horns, which can provide some moderate protection.

### Achieving high sound quality

We recommend using microphones with high signal-to-noise ratios whenever possible ^4^. To date, the Invensense ICS-40720 element has the highest specified signal-to-noise ratio (70 dB) among our MEMS microphones, and the PUI audio AOM-5024L-HD-R has a signal-to-noise ratio of 80 dB. At a price point of respectively 2.72 and 2.58 EUR, they are roughly four times more expensive than the Knowles SPU0410LR5H-QB element (0.62 EUR), and the waterproof Vesper VM1000 element (1.58 EUR) is almost three times more expensive. However, all units are so cheap that replacing broken ones would not be an economic consideration, and they represent only a fraction of the price of commercial microphones.

 It appears that our MEMS microphones do not reach the specified signal-to-noise ratios of most commercial audible range recorder alternatives ^3^. However, this gap is closing quickly (Invensense’s latest ICS-40730 has a signal-to-noise ratio of 74 dB), and there is much variation between manufacturers’ specified signal-to-noise ratios due to loosely standardised measurement protocols ^5^: The Knowles SPU0410LR5H-QB was measured to be on par with ECMs that had specified signal-to-noise ratio values of 80 dB (PUI Audio AOM-5024L-HD-R), with the Vesper VM1000 and the Invensense ICS-40720 closely behind.

 Also, microphone signal-to-noise ratio is almost never measured in the ultrasound range. We could only test ultrasound transmission at 40 kHz, although several bat species vocalise well above 100 kHz. However, no affordable, commercial ultrasound emitters are available to our knowledge. According to our measurements, the Vesper VM1000’s signal-to-noise ratio element trails behind its Knowles and Invensense counterparts for recording ultrasound ^5^. However, the Vesper VM1000 element has the advantage that it does not require a high-performance vent or a windscreen when recording bats, and it can thus be easily combined with horns that make up for that shortcoming.

We would like to stress the benefit of using acoustic horns to amplify sound "for free" when using stereo deployments. The horn we tested considerably improved signal-to-noise ratios, essentially transforming average elements into high-quality microphones. In theory, a sound level increase of 6 dB already causes a doubling of the detection distance, and we measured even larger amplifications. The advantage of such horns has seldom been exploited (but see ultrasonic horn of Wildlife acoustics and the Petterson M500 microphone), although the only downside seems to be the loss in directivity. Unfortunately, when combined with the GAW112 acoustic vent, the horn amplification was mostly cancelled. However, horns provide some protection so that the VM1000 elements could be used outdoors without vents when horns are used for deployments lasting only a few days. According to our simulations, it also appeared beneficial to have a 1 mm long and 1 mm wide circular duct after the horn, which corresponds to the hole in the PCB that leads to the acoustic port of the microphone element.

Surprisingly, we did not find a large signal loss when using long cables. Including pre-amplifiers in microphones that have moderately high sensitivity of -38 dB (like some manufacturers do) seems unnecessary, which simplifies microphone design.

Finally, The Sonitor system can make use of differential-output MEMS microphones. These microphones do not directly lead to lower signal-to-noise ratio: for instance, the Invensense ICS-40720 is a differential output microphone which has the same signal-to-noise ration in single-ended operation. However, differential output microphones cancel out certain sources of noise, such as electromagnetic interference, and they boost the signal because of their doubled output, so that less amplification is needed, thus reducing added noise through amplification. Most recent microphones of Wildlife Acoustics use differential output microphones. The Sonitor system already uses differential output microphones such as the Invensense ICS-40720 but does not make use of this feature yet. Swapping the three-pin audio connector for a four-pin connector to accommodate the additional wire would suffice to realise their potential.

### Sonitor microphone designs

**Figure 9.**
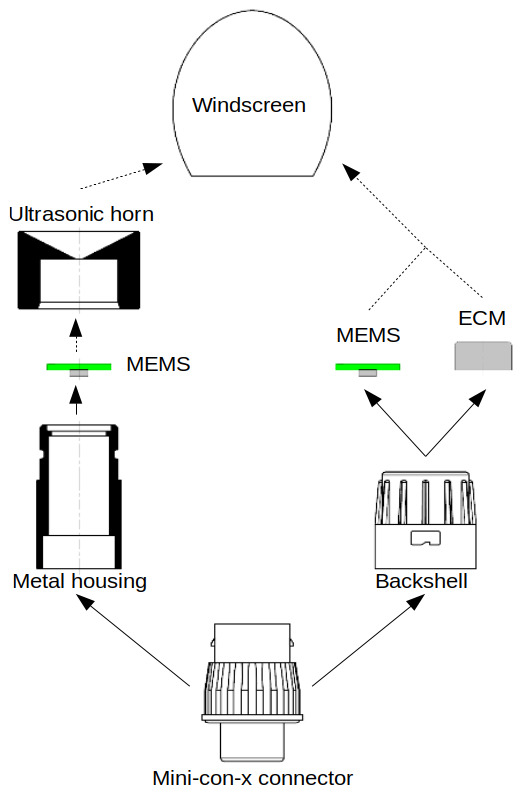
General Sonitor microphone design, showing all parts except the wiring. Optional combinations are shown with dotted arrows.

In the following, we present five microphone designs optimised for different use scenarios ( Table 2). The building instructions are available online ^14^. The basic Sonitor design is flexible ( Figure 9). For the moment, our microphones were tested with the SM2Bat+, a discontinued recorder that allows turning the 2.5 V bias that is usually required for ECMs on and off manually. Unfortunately, more recent recorder models of Wildlife acoustics prevent the use of third-party microphones. The BAR and Swift recorders use the same connector. For Raspberry-Pi based recorders, panel mount connectors (Mini-Con-X reference 6X8X-XSG-XXX) can be installed into the cases to plug our microphones, and MEMS microphones can also be coupled to USB cards ^16^. The Audiomoth uses a MEMS microphone that is directly integrated onto the PCB, but the newest 1.2 version allows the use of external ECM microphones via a 3.5 mm audio socket that users can solder-on themselves. The compatibility of the available recorders with our ECM or MEMS Sonitor microphones is detailed in our online overview ^14^.

***The cheap audible : Bufo***. The Bufo is a low-cost microphone for audible sound surveys only (amphibians, birds, primates), using an ECM. This microphone only consists of an audio connector, wires, an ECM with a GAW325 vent glued onto it, and epoxy glue. The construction of this microphone only requires soldering the capsule to wires, soldering the wires to the connector, gluing the capsule to the connector, and pasting an acoustic vent to the capsule. We recommend using high signal-to-noise ratio microphone elements such as the Primo EM172 ^17^ or the PUI AOM-5024L-HD-R. We found that the Primo EM258 also performs well in the ultrasound range ^5^, but it has a smaller diameter requiring to be carefully glued, and should be compatible only with a trimmed GAW3250408 vent. The capsules can be combined with a windscreen that can be attached using cable ties, and the larger capsules can be combined with the GAW1120509 vent (which has a 9 mm diameter), which has an inner diameter large enough to avoid clogging the microphone holes, and an outer diameter that is smaller than the capsule. The advantage of this design is that it is not necessary to build PCBs or metal housings, however acoustic horns cannot be attached. The design also offers a higher theoretical signal-to-noise ratio at 1 kHz than the current best microphone MEMS.

 It is possible to couple very small capsule microphones such as Knowle’s FG-23629-C36 element, which is often used in bat recorder microphones (SMX-US1 Wildlife acoustics, Batlogger, Petterson) with the Bufo design to achieve a low-cost ultrasound ECM. Indeed, the regular, descending frequency response of that element is desirable ^18^, however it has a very low sensitivity of -53 dB and thus needs strong amplification at the source (by installing high-quality pre-amplifiers inside the housing) to achieve acceptable signal-to-noise ratios for monitoring bats.

***The cheap allrounder: Pavo***. This microphone is similar to the Bufo but it uses the Vesper VM1000 microphone on its PCB with a GAW112 vent glued onto it, and epoxy glue. The Pavo is intended to make full-spectrum recordings from low-frequency amphibian calls up to high-frequency bat calls. The construction of this microphone only requires soldering the ordered PCBs to wires, soldering the wires to the connector, gluing the PCB to the connector, and pasting an acoustic vent to the PCB. The Vesper VM1000 microphone is best suited for this design without a windscreen as it can withstand higher environmental stress due to its piezoelectric design when coupled with the GAW112 vent. This design is not modular – horns cannot be attached – but broken microphone PCBs can be discarded to salvage the connector and install a new PCB.

***The silent one: Otus***. Like its namesake, this is a microphone with a low specified self-noise, enabling high-quality full-spectrum recordings. It is using the Invensense ICS-40720 MEMS element, so it is compatible with a different set of recorders than the Bufo. It also consists of a metal housing enabling horns (for audible sound) to be attached and offering a more sturdy build quality, as well as an audio connector. We originally recommended to use a GAW325 vent for recording audible sound (see article version 1), but we measured much stronger attenuation than specified by the manufacturer at 1 kHz, so that we recommend using the GAW112 vent instead in combination with a windscreen. This microphone element’s differential output can be used with compatible recorders.

***The conventional: Parus***. The Parus uses a tried-and-tested Knowles SPU0410LR5H-QB MEMS element, a GAW112 vent with the necessary windscreen, allowing ultrasound to be recorded, essentially yielding a microphone similar to Wildlife Acoustic's SMM-U1. However, the Parus can also record audible sound and could have higher-quality recordings: The SMM-U1 probably uses the same Knowles FG element as the SMX-U1 that we tested and found to have shorter detection ranges (Darras et al. https://peerj.com/articles/9955). We recommend this configuration when single omnidirectional microphones are required (horns cannot be attached because the windscreen is recommended) and rain is not too frequent as to avoid ultrasound transmission losses due to water-logged windscreens.

***The ultrasonic: Myotis***. The Myotis does not require a wind screen, is more modular than the Parus and better suited for bats. Even though it records the entire sound spectrum, the audible sound interval is recorded slightly less cleanly than with the Knowles SPU0410LR5H-QB or Invensense ICS-40720 elements due to the lower specified signal-to-noise ratio. The microphone uses a waterproof Vesper VM1000 microphone with a GAW112 vent glued onto it. When using the ultrasonic horn to narrow and amplify the pickup area – which is often desirable for bat surveys to focus on flyways – it is recommended to discard the acoustic vent to attain the desired amplification. This combination is particularly useful when doing stereo recordings, where the redundancy of recording with two omnidirectional microphones can be reduced while also increasing the detection ranges. This design without a windscreen enables microphones to dry quickly to record sounds soon after rain. Wind friction is restricted to low frequencies and thus not problematic when recording bats, but it is still possible to attach windscreens in areas prone to wind when low-frequency sound recordings are desired.

***Cost***. We assessed the cost in working time and money of the Sonitor designs at each step of the microphone building process for 100 units ( Table 1). We considered the ordering of individual parts, components assembly, and microphone testing. Our labour estimates are conservative as it can take considerable and variable time for finding suppliers, choosing the design, and setting up of the microphone building. We estimated labour and prices from our own purchases and working time in December 2018, and these prices are representative for Germany and countries with similar supply chains. For the costs of building the PCBs and metal housings and horns, we asked three different suppliers in Germany for quotes and chose the best offer. Prices do not increase proportionally with the number of units due to economies of scale, so that smaller amounts of microphones would pricier, and larger amounts would be cheaper, per unit.

**Table 1.** Cost (EUR) and labor (min) for each step of building the five recommended designs. Prices (as of December 2018) do not increase proportionally with the number of units due to economies of scale. Complete data are available from the Open Science Framework ^14^.

Step	Bufo	Pavo	Otus	Parus	Myotis (with horn)
Buy and adapt 3-pin connectors	467 / 5	467 / 5	467 / 23	467 / 23	467 / 23
Buy wires, epoxy glue, solder iron	50 / 105	50 / 105	50 / 105	50 / 105	50 / 105
Order complete PCBs or ECMs	183 / 10	618 / 20	698 / 20	618 / 20	618 / 20
Order metal housings, horns, windscreens			944 / 15	944 / 15	2133 / 15
Solder wires to PCB and connector	NA / 200	NA / 200	NA / 200	NA / 200	NA / 200
Insert and glue microphone	NA / 100	NA / 100	NA / 100	NA / 100	NA / 100
Glue tube to connector			NA / 100	NA / 100	NA / 100
Purchase and glue acoustic vent	135 / 200	67 / 100	135 /100	67 / 100	67 / 100
Test microphone	NA / 100	NA / 100	NA / 100	NA / 100	NA / 100
**Total for 100 units**	**835 EUR /** **10.3 hours**	**1202 EUR /** **10.5 hours**	**2294 EUR /** **13 hours**	**2146 EUR /** **13 hours**	**3335 EUR /** **13 hours**

**Table 2.** Different microphone designs with their characteristics. Complete data are available from the Open Science Framework ^14^. Costs and assembly times are broken down in Table 1. *this design uses the PUI audio AOM-5024L-HD-R microphone capsule.

Codename	Bufo *	Pavo	Otus	Parus	Myotis (with horn)
**Target sound**	Audible	Full spectrum	Full spectrum	Full spectrum	Full spectrum
**Signal-to-noise ratio in** **dB (1 kHz)**	80	62	70	63	62
**Relative signal-to-noise** **ratio in dB (40 kHz)**	35–38	36–54	57–68	58–64	36–54
**Cost in EUR**	8	12	23	21	33
**Assembly in min**	6	6	8	8	8
**Windscreen**	Recommended	Possible	Required	Required	Possible
**Horn**	Not compatible	Not compatible	Audible horn	Not compatible	Ultrasonic horn or Audible horn

## Future developments

F1000Research allows for article versioning; We welcome prospective co-authors to continue developing our open-source microphone system. Further technological improvements will lead to new, improved microphone elements, and there are many development opportunities:

- We are striving to make the Sonitor system compatible with more recorders. We need to test the microphones on the Audiomoth, Swift, newer Song Meters, and Raspberry-Pi based recorders. Comparisons with commercial microphones could also show how competitive the Sonitor designs are.
- More acoustic vents should be tested to find high-performance acoustic vents that do not reduce ultrasound transmission too much. Alternative products should be found because Gore vents can only be purchased in impractical batches of 1000 from the manufacturer.
- Future Otus microphones could include a newer Invensense microphone (the ICS-40730), which has a higher signal-to-noise ratio of 74 dB. The existing PCB layout should be adapted to the slightly greater microphone dimensions.
- We still need to design light, attachable horns to amplify audible sound.
- The signal loss in even longer cables should be tested, and if substantial, small amplifiers should be designed to compensate for that loss.
- Finally, testing the Sonitor microphones in freshwater systems could reveal new opportunities in that field.

## Data availability

### Underlying data

Raw data for microphone assessment are available on OSF in folder: Microphone assessment. Data for different cable lengths, cable drying, cost and labor, and transmission are available in the indicated csv files.

DOI: https://doi.org/10.17605/OSF.IO/HEZKW ^14^.

### Extended data

**Expanded microphone building instructions are available on OSF in folder: Building instructions.**

DOI: https://doi.org/10.17605/OSF.IO/HEZKW ^14^.

All data are available under the terms of the Creative Commons Zero "No rights reserved" data waiver (CC0 1.0 Public domain dedication).
