# Supplementary material for: Assembling cheap, high-performance microphones for recording terrestrial wildlife: the Sonitor system
Source: F1000Res. 2021 Feb 19;7:1984. Originally published 2018 Dec 28. [Version 3] doi: 10.12688/f1000research.17511.3 (PMC6338251; doi:10.12688/f1000research.17511.3)
Supplement: Peer review history

# Peer review: Reviewer response for version 2

**DOI:** 10.5256/f1000research.23009.r56232

**Published:** 2019-11-15

**Authors:** Sarab S. Sethi

**Affiliations:** 1 Department of Life Sciences, Dyson School of Design Engineering, Imperial College London, London, UK

This is all fine by me now. I thank the authors.

Is the rationale for developing the new method (or application) clearly explained?

Yes

Is the description of the method technically sound?

Yes

Are the conclusions about the method and its performance adequately supported by the findings presented in the article?

Yes

If any results are presented, are all the source data underlying the results available to ensure full reproducibility?

Yes

Are sufficient details provided to allow replication of the method development and its use by others?

Yes

Reviewer Expertise:

NA

I confirm that I have read this submission and believe that I have an appropriate level of expertise to confirm that it is of an acceptable scientific standard.

# Peer review: Reviewer response for version 1

**DOI:** 10.5256/f1000research.19151.r42345

**Published:** 2019-01-17

**Authors:** Catharina Karlsson

**Affiliations:** 1 Department of Biological Science, National University of Singapore, Singapore, Singapore

This study outlines three open-source hardware solutions to microphones for bioacoustics monitoring. The authors justly acknowledge the lack of transparency from current manufacturers of the technical details of the components, especially the microphone element. Another important note is the decrease in cost in comparison to off-the-shelf products which as the authors note, are not only expensive but difficult and impractical to get repaired. Considering the increasing use of open-source hardware in the bioacoustic field this article is a very nice addition to allow practitioners an easier time of assembling their own equipment.

 Even though this is a nice article, it is a bit difficult to follow at times and it doesn’t flow that well. The introduction is good, but I recommend the authors to look through the structuring of the methods and the results section.

 The justification for only testing MEMS elements is not thorough enough as it is not compared to the other types of elements that are on the market. What are the benefits of using a condenser element for example?

 For the sentence below (methods section) I think it is better to just state the representative frequencies rather than giving readers (especially inexperienced ones) the impression that these are representative frequencies for those taxonomic groups (amphibians and birds go above 10kHz depending on species for example). Especially as it is stated in another section that insects can also hear sound up to ultrasonic frequencies.

 "1 kHz (birds and amphibians), 10 kHz (insects), and 40 kHz (bats)"

 The English need to be proof read up throughout, three examples follow (especially in the methods section, I think it makes it a bit stodgy and difficult to follow).

 “This microphone would be intended mainly for bats.” reads better as “This microphone is intended for bat recordings”.

 “This microphone is the cheapest, simplest, and, like its namesake, ugliest design.”

 Sentences like this one can be cleaned up a bit, *Bufonides *are not ugly (you just have not looked closely enough), and there are better words to use than ugly (i.e. basic design, rough etc).

 “It is thus preferable to reflow-solder MEMS elements to printed circuit boards, which can be made in electronic laboratories or workshops equipped with reflow ovens.”

 Sentences like this one does not make a lot of sense (after the comma), I had to re-read several times before I understood what you meant as the way the sentence is structured it could refer to the MEMs element or the circuit board (but you mean neither). I would use a full stop instead of using a comma then re-write the second part to something along the lines of “Reflow-soldering can be performed in reflow equipped electronic laboratories or workshops”.

 The section I was really interested in was to see how the weatherproofing affected the performance of the microphones – however, there is only mention of how it affects the ultrasonic frequencies? It would be nice to see a graph of how the frequencies are affected by different levels of waterproofing (it would even be interesting to see how complete waterproofing, such as a plastic bag compares to vents and no proofing as I’ve worryingly seen that used at times). In general, the result section feels a bit rushed and not developed enough. It was especially difficult to follow, both in the methods and results section, which element and which vent was used where. Occasionally only the vent is mentioned and no element (remind the reader again).

 I think you missed a bit in the discussion, it is worth mentioning that the first time all these things must be sorted out they will take considerable time and I think your estimate of labour is on the low side. It often takes considerable effort to figure out where to source everything the first time – in addition it needs to be highlighted that these cost estimates are for Europe (Germany to be precise, it is in the supplementary information but I do not think it is mentioned in the text), there can be considerable variation depending on where you are based (both lower and higher). The sourcing time is also a labour and it can take considerable time to find suppliers, sort out shipments etc so it should at least get a mention.

 I am pleased that someone has managed to find waterproof vents that come in smaller batches than 10,000 pieces. I also acknowledge the quite comprehensive supplementary material that is attached with more in-depth details of assembly – the article itself is really the tip of the iceberg of the amount of work that has gone into this study. I do believe that comprehensive manuals and instructions like these are a necessity to ensure other people use it. All in all, this is a very nice and timely article and I hope we start seeing more of this kind of work that is written for field scientists coming out.

Is the rationale for developing the new method (or application) clearly explained?

Yes

Is the description of the method technically sound?

Yes

Are the conclusions about the method and its performance adequately supported by the findings presented in the article?

Yes

If any results are presented, are all the source data underlying the results available to ensure full reproducibility?

Yes

Are sufficient details provided to allow replication of the method development and its use by others?

Yes

Reviewer Expertise:

Acoustic Ecology

I confirm that I have read this submission and believe that I have an appropriate level of expertise to confirm that it is of an acceptable scientific standard.

# Response

**Published:** 2019-10-07

**Authors:** Kevin Darras

**Affiliations:** University of Goettingen, Germany

Thank you for taking your time to critically evaluate our article. We respond to your review comments below but please note that there are extensive additional improvements throughout, outlined in the “Changes from the previous version” text.

 You will see that the methods and results have been re-structured and re-written in parts. Hopefully this meets your expectations.

 In this new version, we acknowledge the benefits of using ECMs and propose a new design based on ECMs (re-named Bufo). We also discuss their characteristics versus MEMS elements.

 We acknowledge that the frequencies we tested are only approximately representative but would like to continue pointing out that they stand for different taxa: “However, terrestrial wildlife sounds span frequencies from 20 Hz to 200 kHz, so we measured the transmission across three frequencies that can roughly be assigned to different taxa: 1 kHz (birds and amphibians), 10 kHz (insects), and 40 kHz (bats)“. These point frequencies should be representative of nearby frequencies as abrupt changes of acoustic properties along the frequency range are physically very unlikely.

 Thank you for pointing out English language mistakes, we corrected them accordingly.

 Your question as to how the lack of waterproofing affects microphone performance was addressed in our new long-term test: microphones break, even the water-proof ones. We do not recommend using plastic bags but since this is what many users have with the Audiomoth, we added this: “Since the Audiomoth was introduced, hundreds of these recorders are protected simply by placing the recorder inside a ziplock bag, which can protect the equipment some time, but severely impacts ultrasound transmission, while frequencies in the audible range are better preserved (7).” We reference a forum post by myself where we field-tested different protection levels. But to keep up with the latest improvements, we added: “Fortunately, a new weatherproof case that uses acoustic vents has recently be developed (8).“

 We underlined that our labor estimates are conservative: “Our labour estimates are conservative as it can take considerable and variable time for finding suppliers, choosing the design, and setting up of the microphone building.” and acknowledge the local context: “ […] these prices are representative for Germany and countries with similar supply chains”

# Peer review: Reviewer response for version 1

**DOI:** 10.5256/f1000research.19151.r42344

**Published:** 2019-01-16

**Authors:** Holger Klinck

**Affiliations:** 1 Bioacoustics Research Program, Cornell Lab of Ornithology, Cornell University, Ithaca, NY, USA

This is a very interesting paper which provides very useful information for researchers in the field of terrestrial bioacoustics, especially those involved in remote passive acoustic monitoring efforts.

 General comment:

 The English in the present manuscript requires improvements. Please carefully proof read and spell check the manuscript to eliminate existing grammatical errors. For example, “ *As transducers of mechanical energy into electrical signals*” is not proper English. Another example is “ *Commercial microphones are relatively expensive, specialized on particular taxa, and often have opaque technical specifications.*” Specialized on should be replaced with specialized for. Also, technical specifications cannot be opaque. This sentence needs to rephrased. For example: Technical specifications on the microphones are often not publicized. Language issues like these exist throughout the manuscript and need to be addressed.

 A few detailed comments:

 Which MEMS type is being used in the recommended designs? Sounds like the Bufo is based on the Vesper 1000 MEMS but it is not mentioned which MEMS was used for the Otus and Myotis.

 What really should be included in the manuscript are frequency response curves for the various designs indicating the sensitivity across the entire frequency range of interest. For example, the gain of the horns will be frequency dependent and alter the frequency response of the actual MEMS. A single frequency test is informative but doesn’t provide enough information. This is especially true for frequencies in the 50-100 kHz range.

 The authors emphasize the importance of the microphone’s SNR. The MEMS mics used in the designs feature SNRs between 60 and 70 dB. However, most of the recording system listed in Table 1 feature mics with a SNR of 80 dB. The authors should include talk about these differences in the discussion section.

 In addition, microphone sensitivity is also an important parameter. How do the selected MEMS mics differ in sensitivity (and compare to the mics listed in Table 1)? Again, a comparative frequency response curve would answer many of these questions.

 Most MEMS these days can be wired differentially or single-ended. Differential outputs are typically lower noise and in case of the MEMS and increase the sensitivity. Is this something which could be accommodated in your design? Should this be considered?

 Many autonomous systems aim for low power consumption. How do MEMS compare to traditional mic designs in that regard?

 BTW, TDK recently released the ICS-40730 MEMS with a SNR of 74dB. To my knowledge this is currently the MEMS with the best SNR.

Is the rationale for developing the new method (or application) clearly explained?

Yes

Is the description of the method technically sound?

Yes

Are the conclusions about the method and its performance adequately supported by the findings presented in the article?

Yes

If any results are presented, are all the source data underlying the results available to ensure full reproducibility?

Yes

Are sufficient details provided to allow replication of the method development and its use by others?

Yes

Reviewer Expertise:

NA

I confirm that I have read this submission and believe that I have an appropriate level of expertise to confirm that it is of an acceptable scientific standard.

# Response

**Published:** 2019-10-07

**Authors:** Kevin Darras

**Affiliations:** University of Goettingen, Germany

Thank you for critically reviewing our article. We respond to your review comments below but please note that there are extensive additional improvements throughout, outlined in the “Changes from the previous version” text.

 We corrected all English mistakes that we could spot and also those that you pointed out, thank you. We did not fully write out the microphone model names throughout the manuscript, we do that now.

 We do not have the necessary hardware for carrying out frequency sweeps in the ultrasound range, so we focused on the three representative frequencies of 1, 10, and 40 kHz.

 We discuss the fact that our MEMS have lower specified signal-to-noise ratios than some of the other recorders’ microphones now: “It appears that in theory, our microphones do not reach the specified signal-to-noise ratios of most commercial audible range recorder alternatives (4). However, there is much variation between manufacturers’ specified signal-to-noise ratios due to loosely standardised measurement protocols, and the Knowles SPU0410LR5H-QB was measured to be on par with ECMs that had specified signal-to-noise ratio values of 80 dB (PUI Audio AOM-5024L-HD-R), with the Vesper VM1000 and the Invensense ICS-40720 following closely behind (unpubl. data). Also, microphone signal-to-noise ratio is also almost never measured in the ultrasound range. According to our measurements, the Vesper VM1000’s signal-to-noise ratio element trails behind its Knowles and Invensense counterparts for recording ultrasound (unpubl. data).” This is based on our study which is in review elsewhere (for 7 months), but has been provided as additional material for the reviewers to consider.

 We also detail microphone sensitivity now: “All three MEMS elements have a typical sensitivity of -38 dB and thus require relatively strong amplification from the recorder for soundscape recordings.”

 We also mention differential output microphones in the discussion. We mention the ICS-40730 microphone (which has a bigger form factor) with a 74 dB signal-to-noise ratio now and encourage peers to design a PCB for it.

# Peer review: Reviewer response for version 1

**DOI:** 10.5256/f1000research.19151.r42343

**Published:** 2019-01-15

**Authors:** Sarab S. Sethi

**Affiliations:** 1 Department of Life Sciences, Dyson School of Design Engineering, Imperial College London, London, UK

I enjoyed reading this paper, in particular the thorough nature of the methodology to test various configurations and their effect on amplification, directionality and quality of the audio signal recorded by the microphones. Furthermore, the three clear recommended designs will be particularly useful for ecologists to immediately start incorporating this research into their projects. Commercially available microphones rarely appreciate their weaknesses with such honesty, and as such it’s difficult to find the correct solution for each situation without specialist knowledge.

 I only have a small number of comments as outlined below, but generally I believe this paper is welcome, and should add to the growing appetite for high quality engineering in the field.

 General

- The structure of the paper didn’t quite flow from the Results to Discussion sections. On page 7, in the text under ‘Cost’ and in Table 1 mention is made to Bufo, Otis and Myotis whilst full descriptions of each of these configurations is only given a lot later. I’d recommend moving the recommended designs to the end of the Results section of this paper rather than Discussion
- The lowest frequency tested in all your examples is 1kHz, however you also mention that audible range goes as low as 20Hz. Many terrestrial mammals vocalise with fundamental frequencies under 1kHz (e.g. gibbons, elephants). Ideally we could see results starting at 100Hz or so, or if not this limitation should be made clear in the text

Introduction

- “microphone signals are filtered at the source only for commercial reasons, to enable either bird or bat recordings and sell multiple specialised products” – I can believe this, but would like to see a citation

Methods

- You only compare MEMS microphones for well justified reasons. However, I would still like to see this mentioned more clearly in the introduction or even the abstract. Some mention of Electret Condenser Microphone (ECM) drawbacks would be appreciated
- “This connection form is commonly used in most autonomous sound recorders” – which ones?

Results

- “The vent-only and 6mm horn configurations were affected by wind friction noise at up to 3 kHz, greatly masking the 1 kHz test tones” – I expect this will be a lot worse for lower frequencies I suggested testing above?

Discussion

- It is possible to keep windscreens mostly dry – if they are mounted under a sheltered place (e.g. under a solar panel in Sethi et. al.). They will still get wet, but nowhere near the submerged drenching described here. If this is possible, would this change recommendations?
- Table 1: give per unit costs too please
- “We would like to stress the benefit of using acoustic horns to amplify sound “for free”.” – but later you appreciate the added directionality. This is very important in mono setups and definitely not a free amplification

Is the rationale for developing the new method (or application) clearly explained?

Yes

Is the description of the method technically sound?

Yes

Are the conclusions about the method and its performance adequately supported by the findings presented in the article?

Yes

If any results are presented, are all the source data underlying the results available to ensure full reproducibility?

Yes

Are sufficient details provided to allow replication of the method development and its use by others?

Yes

Reviewer Expertise:

Autonomous ecosystem monitoring, bioacoustics, time series analysis

I confirm that I have read this submission and believe that I have an appropriate level of expertise to confirm that it is of an acceptable scientific standard, however I have significant reservations, as outlined above.

# Response

**Published:** 2019-10-07

**Authors:** Kevin Darras

**Affiliations:** University of Goettingen, Germany

Thank you for your appreciation of our work. We respond to your review comments below but please note that there are extensive additional improvements throughout, summarised in the “Changes from the previous version” text.

 We improved the flow of the paper: we moved the costs section into the discussion, as it did not fit well within the assessment of the acoustic properties of the microphones, and thus avoided to show results about designs that were not presented yet. We integrated the Sonitor designs better into the discussion to show that they are derived from our results. We also added a summarising paragraph to the start of the discussion to announce our structure better.

 We clarified why we did not assess acoustic performance below 1 kHz: “Since we did not have access to dedicated anechoic rooms, all tests were conducted outdoors, and we also refrained from using test tone frequencies below 1 kHz, as lower-frequency anthropogenic noise was constantly present.”

 We were unsure whether we should specifically mention that Wildlife Acoustics ultrasonic microphones filter audible signals using PCBs integrated into their housings. Their component microphone elements are actually well-suited by design for the audible range as well and we cannot think of any technical reason why this is done; the commercial reason might be to create multiple product niches. We now write in a less derogatory but more specific way: “In some cases, audible sound frequencies are filtered at the source to enable only bat recordings although the underlying microphone elements record the entire acoustic spectrum from amphibians to bats (Wildlife Acoustics, e.g. SMX-U1, SMM-U2 microphones).”

 We now propose a new design using an ECM (re-named Bufo as it focuses on audible sound) and integrated the ECM concept into the article.

 We specify which recorders use the Mini-Con-X connectors: “This connection form is commonly used in most autonomous sound recorders (Wildlife Acoustics and Frontier Labs recorders, Swift, Arbimon)”

 Indeed wind noise will affect interest sounds below 1 kHz even stronger, we mention this now: “However, detectability of target sounds below 1 kHz should be even more negatively affected.”

 Now we also recommend a conventional microphone design with a windscreen - which is actually very similar to the one we use in the field – and acknowledge and cite that solar panels can also protect microphones from rain: “It is also possible to reduce the rain ingress by blocking it with shelters or solar panels (6), although this can block sounds of interest coming from above the microphone to some extent.“

 We choose not to include the per unit cost in Table 1, as this is an unrealistic number for ecological studies, and also because of the following reason: “Prices (as of December 2018) do not increase proportionally with the number of units due to economies of scale.” Thus, prices are higher for lower amounts and lower for higher amounts, and we mention this in the discussion too now.

 We clarify that the amplification of horns comes “for free” now with this amendment: “[…] when using stereo deployments“. Indeed, we stated before: “[microphones] can be used without or with horns to narrow and amplify the pickup area […] This combination is particularly useful when doing stereo recordings, where the redundancy of recording with two omnidirectional microphones can be reduced while also increasing the detection ranges.”
